# Deformation-Tailored MoS_2_ Optoelectronics: Fold-Induced Band Reconstruction for Programmable Polarity Switching

**DOI:** 10.3390/nano15100727

**Published:** 2025-05-12

**Authors:** Bo Zhang, Yaqian Liu, Zhen Chen, Xiaofang Wang

**Affiliations:** 1 Department of Physics, Shanghai Normal University, Shanghai 200234, China; 2 School of Arts and Sciences, Shanghai Dianji University, Shanghai 201306, China; 3 State Key Laboratory of Infrared Physics, Shanghai Institute of Technical Physics, Chinese Academy of Sciences, Shanghai 200083, China; 4 School of Microelectronics, Shanghai University, Shanghai 200444, China

**Keywords:** MoS_2_, band structure engineering, structure engineering, electric field modulation

## Abstract

This study proposes an innovative design strategy for molybdenum disulfide (MoS_2_) optoelectronic devices based on three-dimensional folded configurations. A “Z”-shaped folded MoS_2_ device was fabricated through mechanical exfoliation combined with a pre-strain technique on elastic substrates. Experimental investigations reveal that the geometric folding deformation induces novel photocurrent response zones near folded regions beyond the Schottky junction area via band structure reconstruction, achieving triple polarity switching (negative–positive–negative–positive) of photocurrent. This breakthrough overcomes the single-polarity separation mechanism limitation in conventional planar devices. Scanning photocurrent microscopy demonstrates a 40-fold enhancement in photocurrent intensity at folded regions compared to flat areas, attributed to the optimization of carrier separation efficiency through a pn junction-like built-in electric field induced by the three-dimensional configuration. Voltage-modulation experiments show that negative bias (−150 mV) expands positive response regions, while +200 mV bias induces a global negative response, revealing a dynamic synergy between folding deformation and electric field regulation. Theoretical analysis identifies that the band bending and built-in electric field in folded regions constitutes the physical origin of multiple polarity reversals. This work establishes a design paradigm integrating “geometric deformation-band engineering” for regulating optoelectronic properties of two-dimensional materials, demonstrating significant application potential in programmable photoelectric sensing and neuromorphic devices.

## 1. Introduction

Transition metal dichalcogenides (TMDCs), exemplified by molybdenum disulfide (MoS_2_), have demonstrated tremendous potential in novel optoelectronic devices due to their unique layered structure, tunable bandgap, and exceptional optoelectronic properties [1,2,3]. As a prototypical two-dimensional semiconductor, the band structure of MoS_2_ plays a decisive role in its photoelectric conversion efficiency: monolayer MoS_2_ possesses a direct bandgap (~1.8 eV), while its multilayer counterparts exhibit an indirect bandgap (~1.2 eV) [4,5,6]. Its atomic thickness endows it with remarkable mechanical flexibility [7,8], making it an ideal platform for developing novel flexible optoelectronic devices. Nevertheless, conventional planar MoS_2_ devices remain constrained by the single-carrier separation mechanism at Schottky junctions [9,10], where their photocurrent response intensity [11,12] and spatial modulation capabilities fall short of the requirements for advanced optoelectronic systems.

In recent years, three-dimensional structural design has opened new avenues for functional modulation of two-dimensional materials [13,14,15]. By constructing non-planar geometries such as folds and wrinkles, localized built-in electric fields and band structure reconstruction can be introduced at the nanoscale [16,17,18,19], thereby overcoming the physical limitations of planar devices. Among these configurations, folded structures demonstrate remarkable advantages due to their controllable periodic morphology and unique carrier transport characteristics [13,19,20,21,22,23,24,25,26]. For instance, geometric deformation induced by folding can generate polarized electric fields [27,28], while simultaneously modifying the electronic density of states [29,30], thereby establishing multi-dimensional carrier separation channels. Nevertheless, elucidating the underlying optoelectronic response mechanisms and achieving dynamic modulation remain critical challenges in this field.

**Figure 1:**
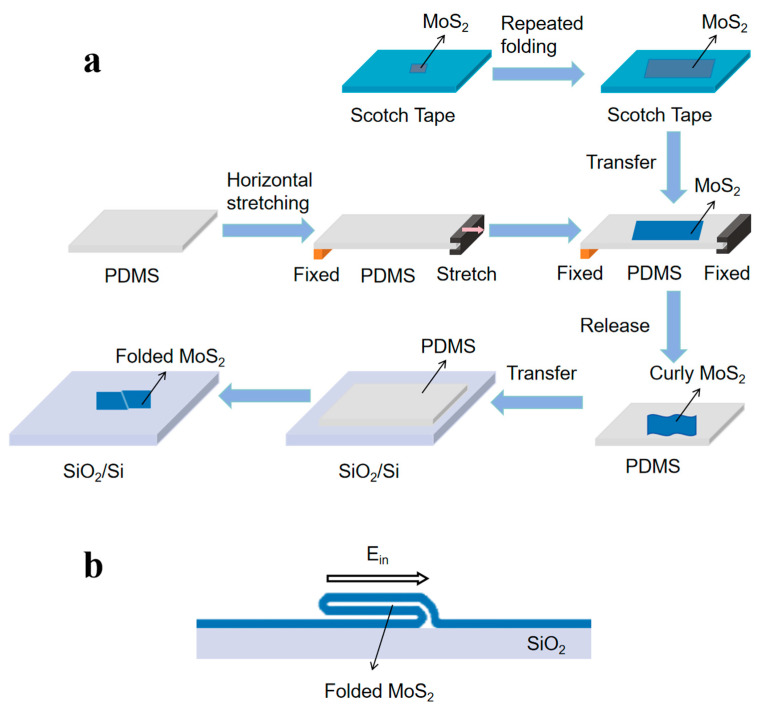
Folded MoS_2_ material preparation. (**a**) Flow chart of material preparation. (**b**) Cross-section diagram of folded MoS_2_ material.

**Figure 2:**
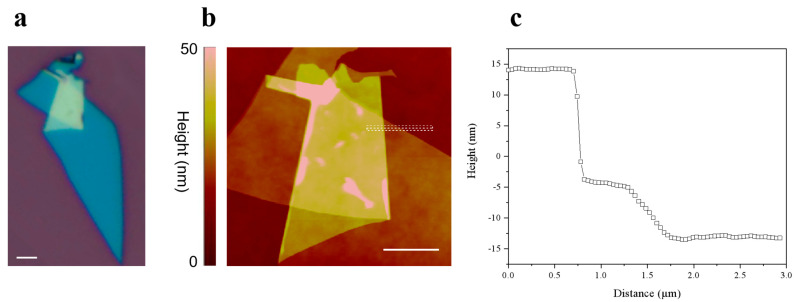
AFM height measurement results of folded MoS_2_ thin films. (**a**) Optical microscopy image of MoS_2_ thin film. Scale bar: 5 µm. (**b**) AFM scan image of MoS_2_ thin film. Scale bar: 2.5 µm. (**c**) Height profile corresponding to the white dashed box in (**b**).

**Figure 3:**
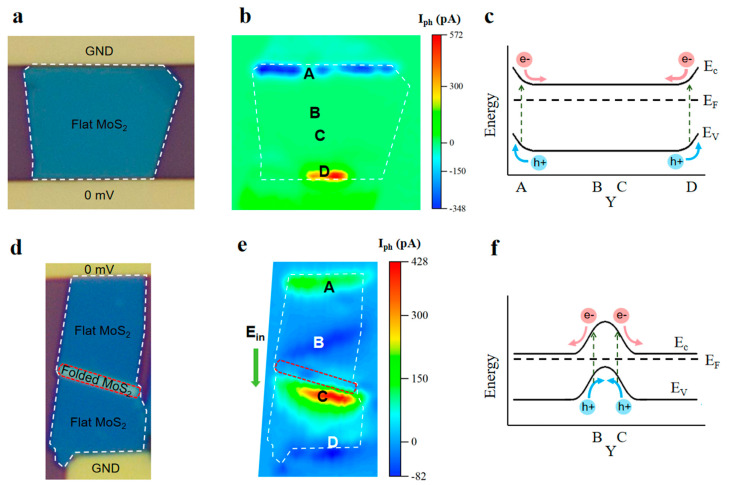
Carrier separation and band structure comparison of flat MoS_2_ optoelectronic devices and folded MoS_2_ optoelectronic devices. (**a**) Optical microscope map of flat MoS_2_ optoelectronic devices. (**b**) Test plots of scanning photocurrent microscopy corresponding to the device shown in (**a**). The area in the white dashed box corresponds to the MoS_2_ channel area in the white dashed box in (**a**). (**c**) Schematic diagram of the band structure corresponding to regions A, B, C, and D in (**b**). (**d**) Optical microscope map of folded MoS_2_ optoelectronic devices. (**e**) Scanning photocurrent microscope test map corresponding to the device shown in (**d**) (dark environment); the region within the white dashed box corresponds to the MoS_2_ channel region within the white dashed box in (**d**); the area in the red dashed box corresponds to the MoS_2_ folded area in the red dashed box in (**d**). (**f**) Schematic diagram of the band structures corresponding to regions A, B, C, and D in (**e**).

**Figure 4:**
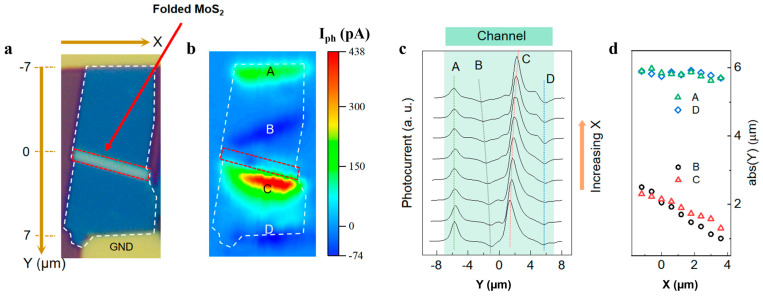
Carrier separation of folded MoS_2_ optoelectronic devices (under indoor white light irradiation). (**a**) Optical microscope map of folded MoS_2_ optoelectronic devices. (**b**) Corresponding scanning photocurrent microscope test map of the device shown in (**a**); the white dotted box corresponds to the MoS_2_ channel area in (**a**), and the area in the red dashed box corresponds to the MoS_2_ folded area in the red dashed box in (**a**). (**c**) The photocurrent distribution and the optical current intensity on multiple lines is extracted in (**b**), where the green, black, red, and blue dashed lines correspond to the optical current intensity in areas A, B, C, and D, respectively. (**d**) Results of coordinate transformation and data processing: change the original X/Y axis and take the absolute value of the new Y axis (original X axis) coordinates to eliminate the direction dependence and enhance the contrast.

**Figure 5:**
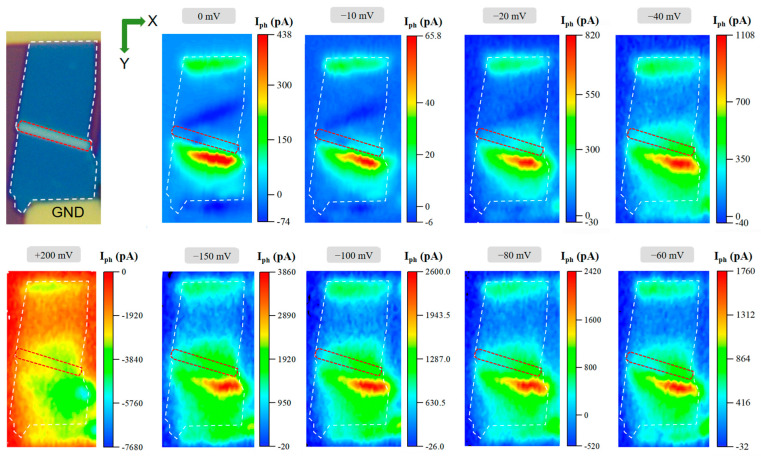
Carrier separation of folded MoS_2_ photoelectric devices under electrical modulation. The photoelectric device was modulated at the voltages of −10 mV, −20 mV, −40 mV, −60 mV, −80 mV, −100 mV, −150 mV, and +200 mV and compared with the test results of the scanning photocurrent microscopy of the device at the voltage of 0 mV, and the region in the red dashed box corresponds to the region in the white dashed channel frame of the optical microscope photograph in the first picture.

This study presents a dual-folded MoS_2_ optoelectronic device design through a three-step fabrication protocol (mechanical exfoliation–stress release–transfer molding; Figure 1), creating a “Z”-shaped structure of the three-dimensional fold in MoS_2_ films (Figure 2). By synergistically integrating geometric folding with band engineering, three critical advancements are achieved: (i) The folded architecture introduces B/C photoresponse zones (Figure 3) beyond conventional Schottky junction regions (A/D), enabling triple photocurrent polarity switching (negative–positive–negative–positive) through coordinated geometry-induced polarization fields and band bending, surpassing the single-polarity limitation of planar devices. (ii) A 40-fold photocurrent enhancement in folded regions compared to flat areas (Figure 4) originates from optimized carrier separation efficiency via three-dimensional configuration-induced pn junction-like built-in fields and piezoelectric potentials. (iii) Dynamic carrier path modulation is realized through external bias, where −150 mV expands positive response regions while +200 mV induces a global negative response, demonstrating co-control of structural regulation and electric field regulation (Figure 5). The demonstrated spatial photocurrent polarity modulation in folded devices not only reveals promising applications in programmable optoelectronics, bio-inspired sensing, and flexible and wearable electronics but also provides mechanistic insights for neuromorphic device engineering.

## 2. Methods

**Sample preparation.** We fabricated folded MoS_2_ devices through mechanical exfoliation of bulk 2H-MoS_2_ (prismatic phase) crystals followed by dry transfer onto SiO_2_/Si substrates (285 nm oxide/500 μm silicon). The critical fabrication steps for folded configurations are schematically illustrated in Figure 1. The process flow includes (i) spin-coating a bilayer photoresist (LOR5A/S1805), (ii) laser direct writing lithography using a Microwriter ML3 system for electrode patterning (Durham Magneto Optics Ltd., Durham, UK), and (iii) sequential deposition of Cr/Au electrodes (15 nm/45 nm) via an EXPLORER-14 electron beam evaporator (Denton Vacuum LLC, Moorestown, NJ, USA), resulting in the final folded device shown in Figure 3d. As control samples, planar devices (Figure 3a) were fabricated under strain-free transfer conditions on PDMS substrates while maintaining identical processing parameters.

**Scanning photocurrent microscopy (SPCM)**. Scanning photocurrent microscopy (SPCM) (Tektronix, Beaverton, OR, USA) measurements were performed using a 520 nm laser diode (Thorlabs, Newton, NJ, USA) with an ITC400 controller. The focused spot diameter of the test is approximately 1 µm.

## 3. Results and Discussion

Figure 1a shows the material preparation process of the folded structure MoS_2_. First, a mechanical stripping method was used to transfer the bulk MoS_2_ crystal to the surface of 3M Scotch blue tape and gradually thinned by a repeated folding stripping technique. The purchased polydimethylsiloxane (PDMS) elastic substrate was removed from its packaging on both sides. Using tweezers, one side was fixed to the edge of the platform with double-sided tape, while the other side was stretched and secured using a clamp to maintain a tensile state. Subsequently, pre-exfoliated multilayer MoS_2_ was transferred onto the surface of the pre-stretched PDMS elastic substrate. After releasing the clamped side of the PDMS and removing the other side from the platform, the tensile stress in the PDMS elastic substrate was completely released. The layered MoS_2_ forms an undulating morphology with a periodic peak–valley fold structure under the in-surface compressive stress. Then, the MoS_2_ film is transferred to the SiO_2_/Si substrate, and the raised peak fold in the film will be inverted to one side, forming a “Z” shape geometry, whose cross-section features are shown in Figure 1b, yielding the double-folded few-layer MoS_2_.

The MoS_2_ thin films prepared via the process illustrated in Figure 1 (optical microscopy image shown in Figure 2a) were subjected to atomic force microscopy (AFM) characterization. The AFM scan of the folded region (Figure 2b) revealed a height difference of 27.3 nm between the folded area and the substrate. In contrast, the smooth uniform layer exhibited a height difference of 9.1 nm relative to the substrate (Figure 2c). The threefold difference in height (27.3 nm vs. 9.1 nm) conclusively confirms a “Z”-shaped folded configuration in the material, consistent with the structural characteristics of vertically stacked folding layers.

Based on the folded structure few-layer MoS_2_ material shown in Figure 1, we prepared photoelectric devices with a dual-folded configuration by a standard lithography and metal evaporation process (Figure 3d). To characterize its spatially resolved photoelectric properties, scanning photocurrent microscopy (Scanning Photocurrent Microscopy, SPCM) was systematically tested at room temperature. The experimental system is equipped with a focused laser source with a wavelength of 520 nm and a spot diameter of 1 µm. The cooperative control of the confocal microscopic system and the XYZ nanotranslation platform achieved point-by-point scanning with submicron accuracy in the two-dimensional plane of the device surface. After the synchronously recorded real-time photocurrent signal is processed by the lock-in amplifier system, the spatial photocurrent distribution map of the dual-fold MoS_2_ device is constructed (Figure 3e). Four characteristic photocurrent response regions are formed on the surface of the folded MoS_2_ device (Figure 3e mark A–D). The A/D region is located at the MoS_2_/Au interface, and its positive/negative photocurrent signal originates from the directional separation of photocarriers driven by the Schottky barrier. This phenomenon also exists in the corresponding interface region of flat structural devices (Figure 3b-A/D). It is worth noting that the B/C region presents a back-to-back arrangement centered on the folded region, and its reverse photocurrent polarity (B negative/C positive) indicates that the photocarriers generated at these two positions will flow in opposite directions. The comparative analysis shows that spatial photocurrent mapping reveals that the area (B/C) near the folded regions achieves comparable responsivity to Schottky junctions (A/D), whereas conventional planar counterparts show noise-floor responses in analogous regions (Figure 3b). This significant enhancement stems from deformation arising from the double-folded structure, which is more conducive to the collection of photogenerated carriers.

Figure 3c,f shows the band structure evolution of the flat and folded configurations, respectively. In the traditional Schottky junction area (A/D position), the spatial separation mechanism of the interface dominates the interface: the conduction band bend drives the photoexcited electron transport to the channel region, while the valence band bend causes hole migration to the metal electrode (Figure 3c). These unidirectional transport characteristics correspond to the negative photocurrent in zone A and the positive response of zone D. It is worth noting that the folded configuration shows unique band engineering characteristics in the B/C interface area (Figure 3f). The band bending caused by in-plane deformation generates a built-in electric field, which leads to bipolar reconstruction of a carrier migration path: a negative response is formed in B, while a positive response is established in C. The observed difference in photoresponse between regions B and C arises from the asymmetric folding geometry of the material, which induces distinct scattering intensities as carriers traverse the folded boundary. As shown in the structural schematic of Figure 1b, the left interface demonstrates significantly higher surface roughness compared to the right. This pronounced topological contrast results in markedly stronger scattering effects during carrier transport across the left interface, thereby fundamentally governing the asymmetric optoelectronic characteristics observed between these regions. The experimental results show that the Schottky barrier and material deformation synergy jointly construct the corresponding complex photoelectric response system and realize the tertiary switching (negative–positive–negative–positive) of the photocurrent polarity between the A-B-C-D characteristic regions. This characteristic provides a new paradigm for the development of reconfigurable optoelectronic devices.

Figure 4 reveals the intrinsic correlation mechanism of the microtopography and photoelectric response properties of dual-folded MoS_2_ devices. The optical microscopy image in Figure 4a clearly shows the unique “Z” dimensional configuration of the folded area, which is three times as high in the vertical direction as the flat area. The scanning photocurrent imaging contrast experiments in Figure 3 reveal a unique photoelectric behavior of the folded structure compared to the conventional flat structure. Under the broad spectrum of white light irradiation (Figure 4b), the overall photocurrent of the device is increased compared with the dark conditions (Figure 3e), which is closely related to the bandgap modulation effect caused by the deformation generated by folding. Theoretical calculations show that due to the local deformation generated by the folding structure, while the band gap of MoS_2_ decreases [13], the energy required for the electron transition from the valence band to the conduction band decreases, and the response wavelength range widens.

The quantitative distribution of photocurrents on multiple lines extracted from Figure 4b (Figure 4c) shows a strong spatial heterogeneity feature. Near the folded region, the photocurrent peak achieves a 40-fold enhancement factor compared to the adjacent flat region. This super-gain effect can be traced to the structural transformation-induced internal electric field. This strong electric field with a carrier separation barrier of pn-like junctions by band bending improves the carriers’ dissociation efficiency. This strain–photoelectric coupling mechanism provides an important theoretical basis for the development of new flexible optoelectronic devices.

Figure 5 illustrates the carrier separation characteristics of dual-folded MoS_2_ optoelectronic devices under electric field modulation. The experiment shows that the bias voltage has a significant regulation effect on the photocurrent response; with the enhancement of the negative bias (0–150 mV), the positive photocurrent response of the device presents two significant features: (i) the positive response intensity is systematically enhanced; (ii) the spatial distribution range is gradually expanded. In contrast, the negative response region showed only weak fluctuations. It is noteworthy that the abnormality occurs at −10 mV critical bias, and the positive response strength and response region are slightly below the zero bias state.

This regulation mechanism originates from the synergy between the energy band structure change produced by the deformation and the electric field: (i) the local deformation generated by the folding structure reduces the MoS_2_ band gap, and the strong electric field formed by the negative bias further bends the band structure to promote the exciton dissociation; (ii) the polarization electric field potential constructs the carrier migration path and reduces the capture probability of the composite center; (iii) the enhanced built electric field accelerates the carrier directional transport. For the –10 mV anomalous response, we propose a double barrier competition model: at this critical bias, the barrier height formed by the tensile strain is comparable to the electric field driving potential, resulting in an increase in the composite probability of the carrier at the defect site. When the bias exceeds −20 mV, the electric field barrier dominates, and the compound rate drops sharply.

The positive bias (+200 mV) presents a completely different regulatory behavior: the photocurrent response shows an overall negative response. This is due to the reduced bending of the energy band by the forward electric field and the decreased efficiency of photoborne carrier separation. Electrons and holes may be “pushed back” by the electric field to the same region (interface or defects) under illumination, and the composite probability increases significantly. This electric modulation scheme verifies the feasibility of mechanical folding strategy for two-dimensional material band engineering, provides a prototype for the development of “structural regulation + electric field regulation” dual-mode photoelectric devices, lays the foundation for the development of light–machine–electric multi-field regulation intelligent devices, and has an important application prospect in programmable photoelectric response, adaptive sensing, flexible wearable electronic devices, and other fields.

## 4. Conclusions

This study systematically deciphers the synergistic coupling between geometric deformation and optoelectronic response through three-dimensional folded MoS_2_ architectures. We developed a mechanical exfoliation–stress release–transfer molding technique to fabricate “Z”-folded photoelectric devices, overcoming carrier separation limitations inherent to planar configurations. Experimental evidence reveals that folding-induced deformation generates built-in electric fields via band structure reconstruction, creating unconventional photocurrent zones beyond Schottky junctions. This mechanism enables triple polarity inversion (negative→positive→negative→positive) with 40-fold photocurrent amplification in folded regions compared to flat counterparts. Voltage-modulation experiments demonstrate dynamic carrier transport reconfiguration: −150 mV bias expands positive-response domains, whereas +200 mV induces global negative polarity, validating dual-mode control through structural–electrical synergy. Our findings establish a materials design paradigm for flexible optoelectronics and intelligent sensing. The demonstrated “mechanical deformation–electronic characteristics” co-regulation mechanism provides a foundational platform for next-generation adaptive optoelectronic systems.

## Data Availability

All the data that supports the plots within this paper and other findings of this study are available from the corresponding author upon request.
